# Views of the public about Snacktivity™: a small changes approach to promoting physical activity and reducing sedentary behaviour

**DOI:** 10.1186/s12889-022-13050-x

**Published:** 2022-03-29

**Authors:** K. Gokal, R. Amos-Hirst, C. A. Moakes, J. P. Sanders, D. W. Esliger, L. B. Sherar, N. Ives, S. J. H. Biddle, C. Edwardson, T. Yates, E. Frew, C. Greaves, S. M. Greenfield, K. Jolly, M. Skrybant, R. Maddison, N. Mutrie, H. M. Parretti, A. J. Daley

**Affiliations:** 1 School of Sport, Exercise and Health Sciences, Loughborough University, Loughborough, Leicestershire LE11 3TU UK; 2 The Centre for Lifestyle Medicine and Behaviour (CLiMB), School of Sport, Exercise and Health Sciences, Loughborough University, Loughborough, Leicestershire LE11 3TU UK; 3 Birmingham Clinical Trials Unit, University of Birmingham, Edgbaston, Birmingham, B15 2TT UK; 4 Physically Active Lifestyles Research Group (USQ-PALs), Centre for Health Research, University of Southern Queensland, Springfield, QLD 4300 Australia; 5 Diabetes Research Centre, University of Leicester, Leicester General Hospital, Leicester, LE5 4PW UK; 6 Health Economics Unit Institute for Applied Health Research, University of Birmingham, Edgbaston, Birmingham, B15 2TT UK; 7 School of Sport, Exercise and Rehabilitation Sciences, University of Birmingham, Edgbaston, Birmingham, B15 2TT UK; 8 Institute for Applied Health Research, University of Birmingham, Edgbaston, Birmingham, B15 2TT UK; 9 Institute for Physical Activity and Nutrition, Deakin University, Melbourne, Australia; 10 Physical Activity for Health Research Centre, University of Edinburgh, Edinburgh, EH8 8AQ UK; 11 Norwich Medical School, Faculty of Medicine and Health, University of East Anglia, Norwich, Norfolk NR4 7TJ UK

**Keywords:** Snacktivity™, Small bouts, Physical activity, Health behaviour change, Survey, Health messaging

## Abstract

**Background:**

Many people do not meet the recommended health guidance of participation in a minimum of 150–300 min of moderate intensity physical activity per week, often promoted as at least 30 min of physical activity on 5 days of the week. This is concerning and highlights the importance of finding innovative ways to help people to be physically active each day. Snacktivity™ is a novel approach that aims to encourage people to do small, 2–5 min bouts of physical activity ‘snacks’ throughout the whole day, such that they achieve at least 150 min of moderate intensity activity per week. However, before it can be recommended, there is a need to explore whether the concept is acceptable to the public.

**Methods:**

A survey to assess the views of the public about Snacktivity™ was distributed to adult patients registered at six general practices in the West Midlands, UK and to health care employees in the same region.

**Results:**

A total of 5989 surveys were sent to patients, of which 558 were returned (9.3%). A further 166 surveys were completed by health care employees. A total of 85% of respondents liked the Snacktivity™ concept. The flexibility of the approach was highly rated. A high proportion of participants (61%) reported that the ability to self-monitor their behaviour would help them to do Snacktivity™ throughout their day. Physically inactive participants perceived that Snacktivity™ would help to increase their physical activity, more than those who were physically active (OR = 0.41, 95% CI: 0.25–0.67). Approximately 90% of respondents perceived that Snacktivity™ was easy to do on a non-working day compared to 60% on a working day. Aerobic activity ‘snacks’ were preferred to those which were strength based.

**Conclusions:**

The Snacktivity™ approach to promoting physical activity was viewed positively by the public and interventions to test the merits of such an approach now need to be developed and tested in a variety of everyday contexts.

**Supplementary Information:**

The online version contains supplementary material available at 10.1186/s12889-022-13050-x.

## Background

Increasing population levels of physical activity is a public health priority. There is compelling evidence that physical inactivity and high levels of sitting are associated with adverse health outcomes and mortality [1–3]. However, a large proportion of the adult population do not meet the internationally accepted physical activity guidelines of participation in 150–300 min of moderate intensity physical activity per week, or 75–150 min of vigorous intensity physical activity per week, to achieve optimal health. Additionally, guidance states that adults should undertake physical activity to improve their muscle strength on at least 2 days per week and emphasises the importance of reducing time spent sitting each day [4, 5]. The most recent physical activity guidelines have removed the requirement for activity to be in bouts of 10 min or longer [4, 5], with a shift towards emphasising the importance of any physical activity for health. Whilst guidelines now recognise that some movement is better than none, greater emphasis has been placed on the overall physical activity target (i.e. 150–300 min of moderate intensity physical activity per week). However, evidence suggests that most of the adult population do not achieve even 40 min of physical activity per week on a regular basis [6]. For example, a study pooling accelerometer assessed physical activity data from four European countries found that 72% of participants are not achieving the physical activity recommendations based on time spent in moderate to vigorous physical activity (MVPA) in bouts of ≥10 min [6] and only 10–30% of adults participated in strength-based physical activity each week [7].

Whilst public health guidance for physical activity has existed for many years, it has not led to the successful uptake and maintenance of physical activity in the population. Evidence suggests that developing physical activity guidelines and recommendations alone does not lead to health behaviour change [8]. Advising the public to ‘move more’, without providing methods or strategies to help them achieve participation in physical activity is unlikely to lead to sustained health behaviour change [9]. It is clear, that guidance that focuses on the public achieving large behavioural goals for physical activity has not been successful in getting the public sufficiently active, and a paradigm shift in the way physical activity is promoted to the public is therefore needed. There is also a need to reduce sedentary behaviours, particularly given that prolonged low energy sitting is now the norm for most people [10].

An alternative approach to physical activity promotion that may engage and motivate people to be more physically active throughout the whole day [11] is a concept referred to here as Snacktivity™. Rather than encouraging large bouts of activity (e.g. 30 min of physical activity per week), Snacktivity™ focuses on achieving small, but frequent, bouts of MVPA throughout the day, to accumulate at least 150 min of MVPA per week. A physical activity ‘snack’ typically lasts between 2 and 5 min, and examples include: walk-talk conversations, using the stairs as opposed to the lift, squats whilst brushing teeth or seated arm raises when watching the television. An important benefit of Snacktivity™ over current physical activity guidance is that it encourages breaking up passive sitting time throughout the day, and places equal emphasis on both aerobic and strength-based activities. Psychological theory and perspectives acknowledge that gradual building of self-efficacy (confidence to change behaviours) is important for promoting behaviour change [9, 11]. Small changes are easier for people to initiate and maintain than large changes [12], therefore the promotion of small bouts of physical activity through Snacktivity™ may also help develop people’s confidence to try to become regularly physically active. Furthermore, because Snacktivity™ can be completed while participating in other daily tasks, (i.e. doing squats whilst brushing your teeth or jogging on the spot whilst waiting for the kettle to boil) it has the potential to address the most common barrier for physical inactivity, a perceived lack of time [13]. Whilst evidence suggests that small bouts of physical activity are associated with a lower risk of chronic disease [14], it is not yet known if this approach is acceptable or how best to provide resources to help the public integrate Snacktivity™ into their day. The aim of this observational study was therefore to explore the views of the public about the Snacktivity™ concept and to gather data to support further development of this approach.

## Methods

### Design

A cross-sectional survey was conducted to assess the views of the public about Snacktivity™. The survey was distributed to adults (referred to hereafter as patients) registered at six socioeconomically diverse National Health Service (NHS) general practices in the West Midlands, United Kingdom to ensure a diverse mix of responses to the survey. The survey was also advertised to staff employed by a local NHS Community Trust to increase the breadth of the study data. Favourable ethical approval was granted by East Midlands, Leicester South Research Ethics Committee (reference number: 19/EM/037). All participants who completed the survey were offered the opportunity to be entered into a prize draw with the potential to win a high street shopping voucher (30 × 10 GBP each).

### Setting and procedure

NHS patients and employees were invited to take part in the survey between March and July 2020 which coincided with the start of the COVID-19 pandemic. Surveys were completed anonymously unless participants chose to provide the research team with their contact details to to be entered into the prize draw, receive study results or be contacted for future studies related to the project.

#### NHS patients

A search of each general practice’s patient list was undertaken to identify eligible patients. These included those aged 18 to 80 years, and excluded those with a terminal illness, dementia or a severe learning disability, those who had expressed a dissent from taking part in research, or any patients unable to provide consent. From those patients identified as eligible to participate, 1000 were selected at random from each practice and were mailed the study pack by their practice. The study pack contained a leaflet explaining the concept of Snacktivity™ and how it could be used to meet the physical activity recommendations, a patient information leaflet, the study survey including a consent statement, a supplementary Snacktivity™ picture booklet (Supplementary file 1) and a Freepost envelope to return the completed survey. Three of the participating practices sent text message reminders to patients which contained an online link to the study documents and the survey which was hosted using the Online Surveys platform. Due to the COVID-19 pandemic and a change to general practice priorities, three practices were unable to send reminders to patients.

#### NHS employees

The study sample was supplemented by inviting both clinical and non-clinical staff aged 18 years or over employed by an NHS Trust to complete the study survey online. The Snacktivity™ study link was displayed in multiple locations accessed by the Trust’s employees including (but not limited to) desktop screen savers, weekly e-bulletins, electronic staff records and on the Research and Innovation website.

### Study survey

Section one asked participants to rate their likeability of the Snacktivity™ concept and if they believed it would help them to be more physically active throughout the day. Participants were asked to indicate potential barriers/facilitators to Snacktivity™ from a prefilled list of statements and were provided with open text boxes to allow them to elaborate on their responses. Section two asked participants to refer to the supplementary picture booklet which illustrated 30 activity snacks in different settings (Supplementary file 1). Participants were asked to indicate which activity snacks they perceived they would find most enjoyable, those they believed would be easiest for them to build into their daily routine and would be likely to do at home and at work. Respondents were also asked to report which activity snacks they perceived to be least appealing to them. Section three asked participants to report the amount of time they had participated in physical activity and sedentary behaviour across the previous seven days using the General Practice Physical Activity Questionnaire (GPPAQ) [15]. A variation of the sitting question from the International Physical Activity Questionnaire (IPAQ) was also included [16]. We were interested in understanding how technology could be used to support the integration of Snacktivity™ into people’s everyday lives to inform future intervention development. Section four, therefore, gathered participants’ views about wearable physical activity technology and physical activity mobile phone applications (apps) using a combination of Likert scales and open text boxes.

All ten members of the Snacktivity™ Public Advisory Group (PAG) provided substantial input into the development of the survey and study materials. This included ensuring the wording of the survey was accessible, the structure was coherent, and that instructions and guidance to participants were clear. The PAG also provided input to the examples of activity snacks presented in the picture bookelet, ensuring a range of activities with people from diverse backgrounds.

### Sample size

No formal sample size calculation was conducted due to the exploratory nature of this study. We anticipated a survey response rate of 20% in patients. Surveys were sent to 6000 patients, allowing for a response rate of 20% to be estimated within a 95% confidence interval (CI) of +/− 1%, we, therefore, anticipated a response from 1200 patients.

### Data analyses

The GPPAQ tool categorises participants into one of four groups, where active refers to those achieving the recommended guidelines of at least 150 min of moderate intensity physical activity per week. Those participants who were considered as ‘moderately inactive’, ‘moderately active’ or ‘active’ by GPPAQ or ‘active’ by GPPAQ-walk were considered as active. All other participants were considered as inactive [17]. A variation of the sitting question from the IPAQ-Short Form was used to record sedentary time across a 7-day period on both working days and non-working days to differentiate between work and leisure and also account for potential variations in working patterns. Total sitting time across working and non-working days was categorised into three groups (< 6, 6–10, > 10 h/day). Other outcomes included use of trackers and apps, Snacktivity™ likeability, participant preferences for activity snacks, participant perceptions of Snacktivity™ increasing activity, participant perceptions of ease of uptake of Snacktivity™ and participant views of Snacktivity™.

### Statistical analyses

Demographic characteristics were summarised for the total sample (patients and staff) using frequencies and percentages. Physical activity status measured using GPPAQ and GPPAQ-walk, tracker and app use were analysed descriptively using frequencies and percentages. Sitting times were summarised using medians, interquartile ranges and ranges, and dichotomised using frequencies and percentages. Snacktivity™ likeability and perceptions of ease of uptake of Snacktivity™ were presented descriptively using counts and percentages, with odds ratios (and corresponding 95% CIs) produced from ordinal regression models. Univariate models were fitted with demographic (age, gender, employment status and socioeconomic status) and physical activity (physical activity status, sitting time) variables. Multivariate models, including interactions terms were planned but not performed due to missing data. Preferences for activity snacks and views of Snacktivity™ were presented visually using bar charts. Perception of Snacktivity™ increasing activity was presented descriptively using counts and percentages by physical activity status (active, inactive). An odds ratio and corresponding 95% CI was produced from an ordinal regression model. Open text box responses were quantified using content analysis [18, 19].

## Results

### Recruitment and participant demographics

A total of 96,733 medical records across six practices were searched for eligible patients. Of the 41,624 eligible patients, 6000 were identified as potential participants and 5989 surveys were distributed. Eleven surveys were not sent due to missing NHS numbers or incorrect postal addresses. A total of 724 surveys were completed; 558 were completed by patients (response rate of 9.3%) and 166 by healthcare staff. Table 1 provides details of the participant demographics.

**Table 1:** Participant demographics

Participant demographics	All participants *N* = 724
Age (years)	
≤ 40	155 (21%)
41–60	309 (43%)
≥ 61	260 (36%)
Gender	
Male	257 (36%)
Female	462 (64%)
Prefer not to say	5 (< 1%)
Employment status^a^	
Employed	492 (68%)
Unemployed	208 (29%)
Other	24 (3%)
Ethnicity^b^	
White	620 (86%)
Non-white	102 (14%)
Missing	2
	**Patients** ***N*** **= 558**
Socioeconomic Status^c^	
Low	113 (20%)
Medium	101 (18%)
High	343 (62%)
Missing	1

^a^Employed (includes full time paid employment, self-employed/ freelance and part-time paid employment), unemployed (includes retired from paid work, looking after the home/ family and unemployed) and other (includes other, student, sick/ disabled, seasonal part-time job, voluntary work, supply teacher, semi-retired, maternity leave

^b^White (includes white) and non-white (includes Indian, Bangladeshi, Pakistani, Chinese, other Asian, black African, black Caribbean, black other, mixed, Korean, Arabic, Middle Eastern/Kurdish/North Iraq, Sikh Asian, Middle Eastern New Zealand/ Samoan and non-specified other

^c^Socioeconomic status was collected from patient responders only, not staff

### Physical activity

Of the 632 participants who completed the GPPAQ and GPPAQ-Walk, 28% were considered physically inactive. Of the 520 participants who completed the IPAQ sitting questions, 40% reported a total sitting time of > 10 h per day and 43% for 6–10 h per day.

### Views of Snacktivity™

**Fig. 1:**
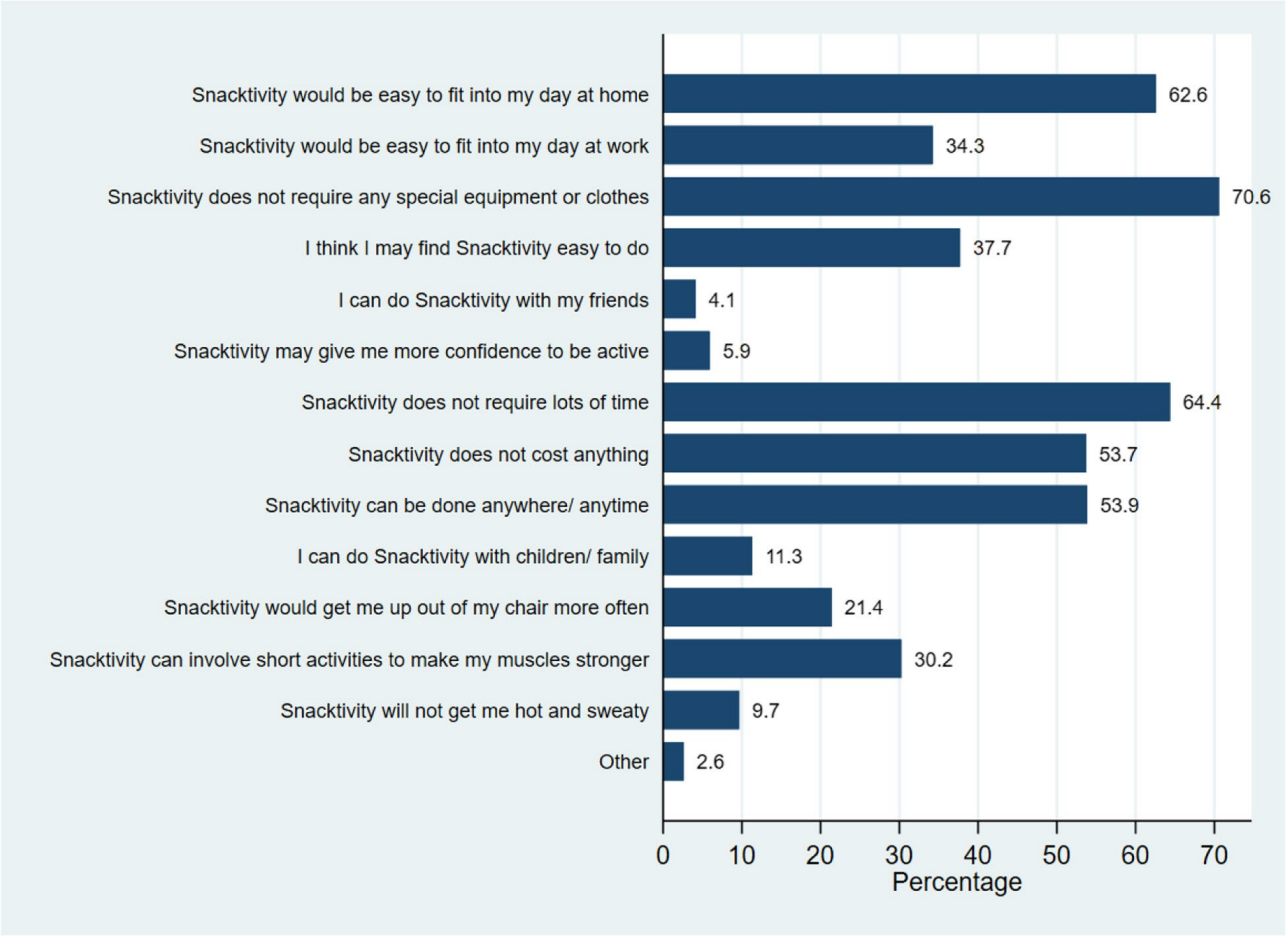
Most liked elements of Snacktivity™ (*N* = 724)

Eighty-five percent of participants liked the Snacktivity™ concept (51% = like it a lot and 34% = like it a bit). From the univariate models (Supplementary file 2), females had higher odds of liking Snacktivity™ than males (OR = 1.68, 95% CI: 1.25–2.25) and those aged ≤40 years had reduced odds of liking Snacktivity™, in comparison to those ≥61 years (OR = 0.66, 95% CI: 0.46–0.96). The most frequently selected reasons for liking Snacktivity™ were that it does not require any special equipment or clothes (71%), that it does not require lots of time (64%) and that it would be easy to fit into the day at home (63%) (Fig. 1). Open text box responses further highlighted positive views towards Snacktivity™ including comments such as “there’s nothing I don’t like about it” and “I love everything about it and think GPs could use it with people who are overweight or need to improve their fitness/muscle strength”.

**Fig. 2:**
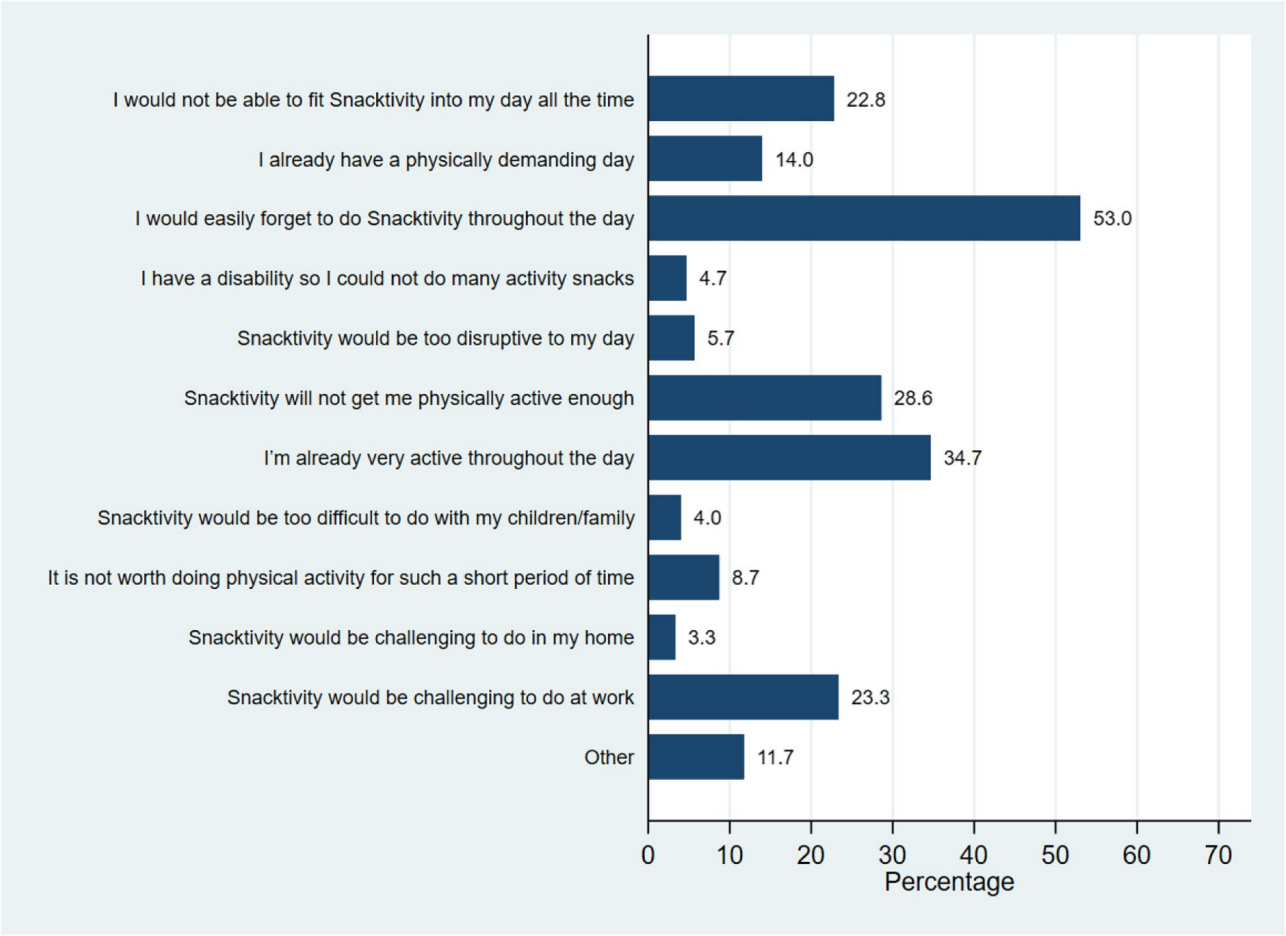
Most disliked elements of Snacktivity™ (*N* = 724)

The concept of Snacktivity™ was disliked by 3% of responders of all participants (2% disliked it a bit and 1% disliked it a lot) and a small proportion (*n* = 2%) did not like the term ‘Snacktivity™’ which was reflected in open text box responses such as ‘The name! It sounds like its more about diet than exercise’. The most frequently selected options for disliking Snacktivity™, among the total sample, were that participants perceived that they would easily forget to do Snacktivity™ throughout the day (53%), that they are already active throughout the day (35%) and that it may not get them active enough (29%), (Fig. 2). These concerns were also expressed in the open text box responses such as ‘I would need support to think of different ideas to keep me motivated’ and ‘I don’t think it will really make much difference to my health/weight’.

**Fig. 3:**
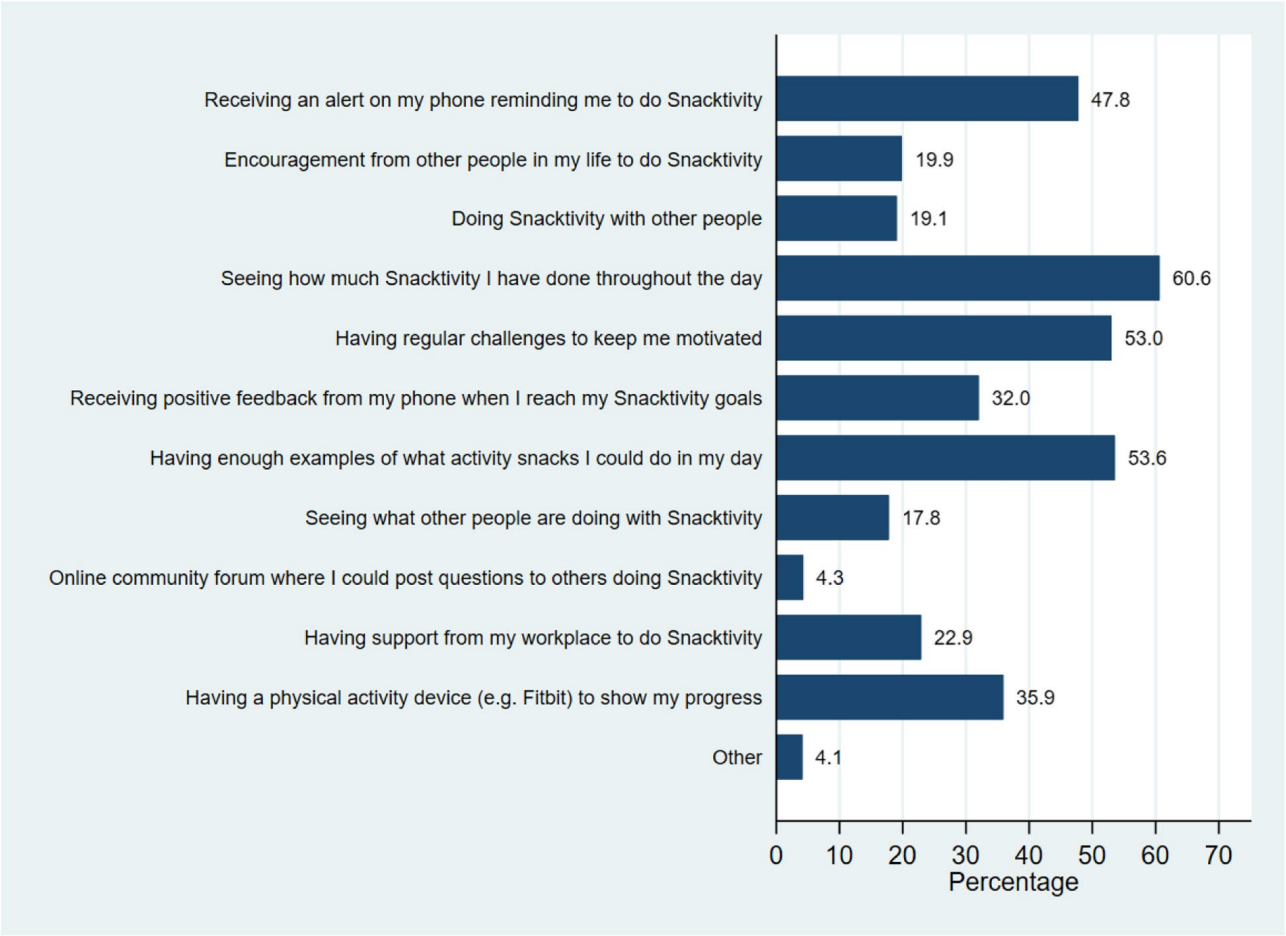
Elements that would help to achieve Snacktivity™ throughout the day (*N* = 724)

When asked what would help participants do Snacktivity™ throughout the day (Fig. 3), the most frequently selected options included: seeing how much Snacktivity™ has been completed throughout the day (61%), having enough examples of activity snacks (54%), having regular challenges (53%) and receiving an alert reminding them to do Snacktivity™ (48%). The ability to share and view other people’s activity, do Snacktivity™ with other people as well as the potential to receive social support were less popular. The most frequently perceived reasons preventing Snacktivity™ throughout the day included forgetting to do Snacktivity™ throughout the day (60%), running out of ideas of what activity snacks to do (31%), not having the motivation to do Snacktivity™ (28%), and feeling self-conscious doing activity snacks around others (20%).

### Perception of Snacktivity™ increasing physical activity

Of the 178 physically inactive participants, 87% agreed that Snacktivity™ would increase their physical activity, as did 74% of the 454 active participants. Inactive participants had higher odds of perceiving that Snacktivity™ would increase their levels of physical activity in comparison to active participants (OR = 0.41, 95% CI: 0.25–0.67).

### Perception of ease of uptake of Snacktivity™ on non-working day

A total of 89% of participants perceived Snacktivity™ to be easy or moderately easy to incorporate into a typical non-working day and only 4% of participants perceived Snacktivity™ to be difficult or moderately difficult to achieve. Estimates obtained from univariate models suggest that physically active participants perceived Snacktivity™ as easier to complete on a non-working day, compared with physically inactive participants (OR = 1.84, 95% CI: 1.26–2.69).

### Perception of ease of uptake of Snacktivity™ on working day

A large proportion of participants (59%) perceived Snacktivity™ to be easy or moderately easy to incorporate into a typical working day, and 20% of participants perceived it as difficult or moderately difficult. Univariate models revealed age, gender, physical activity status and time spent sitting to be associated with perception of ease of uptake of Snacktivity™. Participants aged ≤40 years had lower odds for perceived ease of uptake on a working day compared with those aged ≥61 years (OR = 0.49, 95% CI: 0.31–0.77). In comparison to males, females had lower odds for perceived ease of uptake (OR = 0.59, 95% CI: 0.42–0.82). Physically active participants perceived Snacktivity™ to be easier to incorporate into their day compared to those who were physically inactive (OR = 1.81, 95% CI: 1.26–2.60). This was supported by those who spend less time sitting (< 6 h a day) also perceiving Snacktivity™ to be easier to do compared with those who sit > 10 h a day (OR = 2.07, 95% CI: 1.26–3.40).

### Views regarding different physical activity snacks

**Fig. 4:**
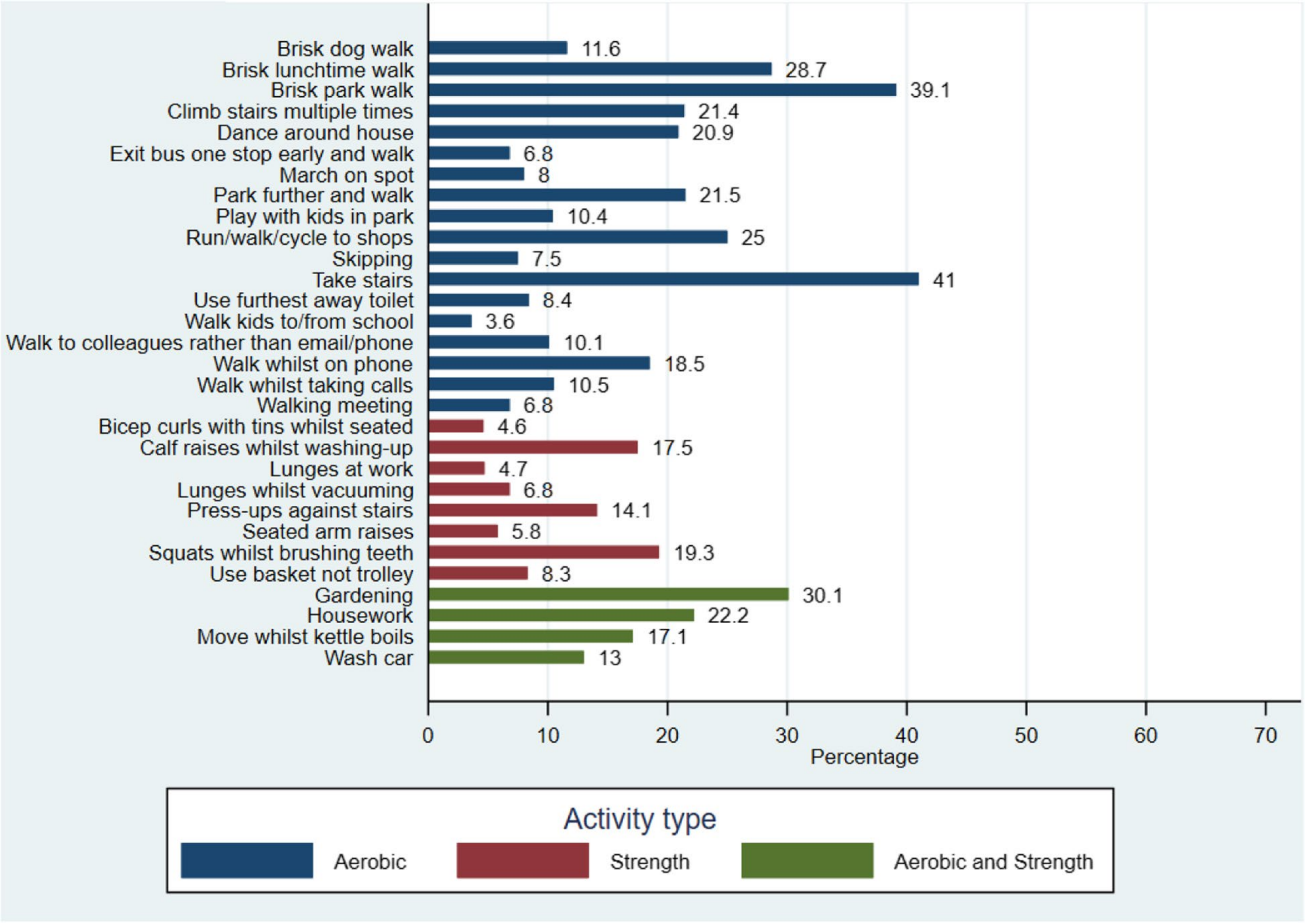
Activity snacks participants perceived they would enjoy (*N* = 724)

**Fig. 5:**
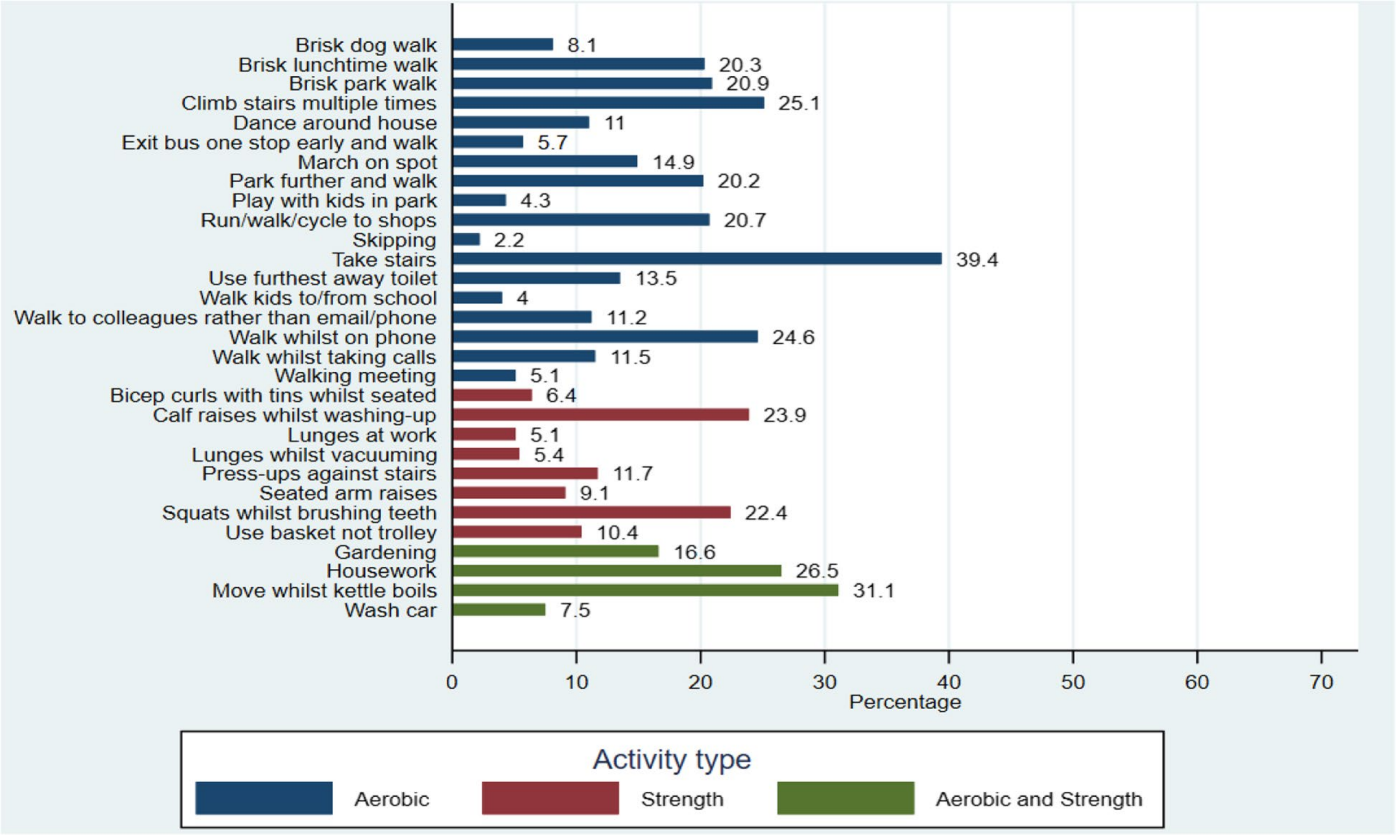
Activity snacks participants perceived they would find easiest to build into their daily routines (*N* = 724)

**Fig. 6:**
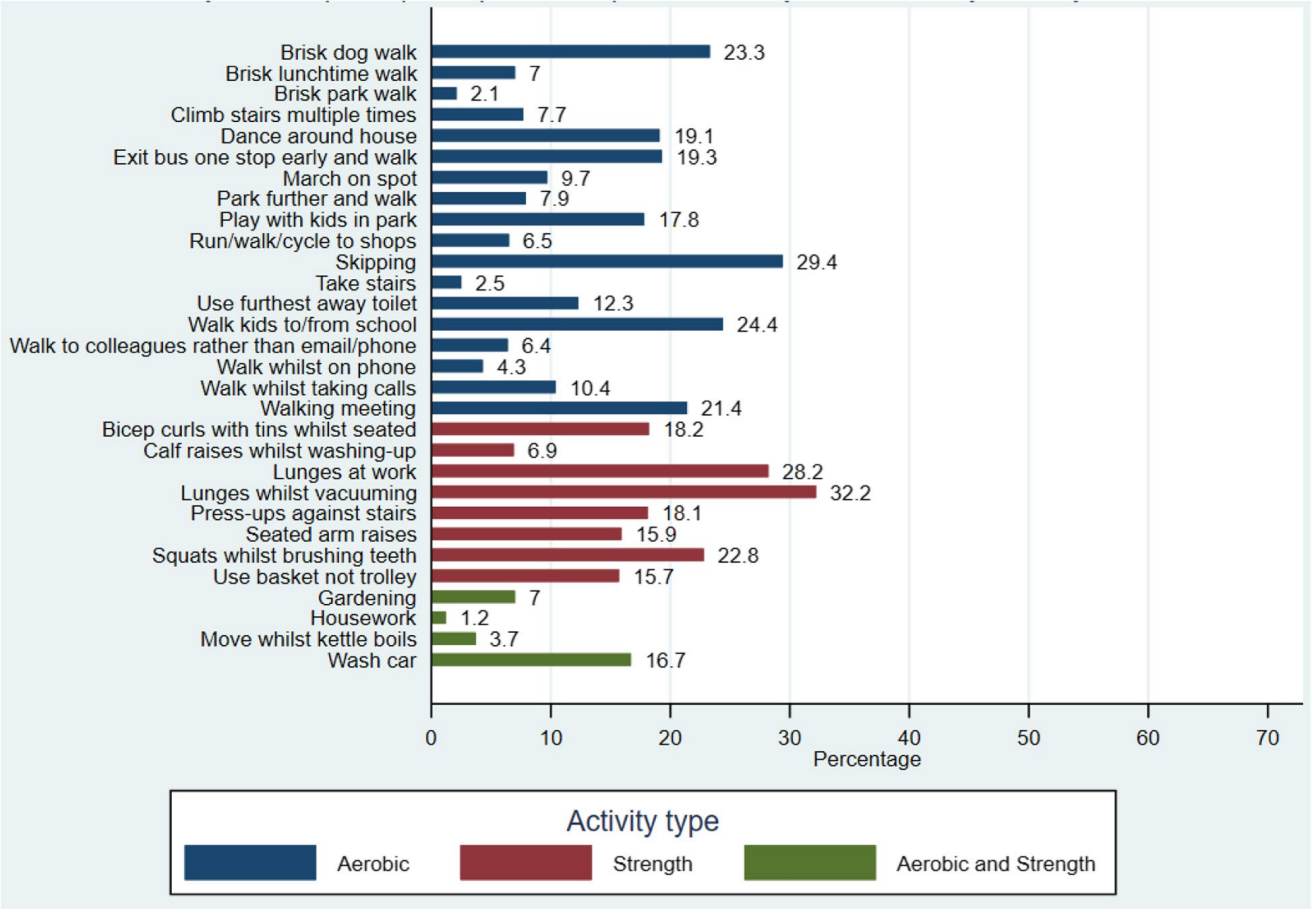
Activity Snacks participants perceived they would be unlikely to do (*N* = 724)

Overall, participants considered aerobic based activity snacks, which involved walking and formed part of their daily routine (i.e. taking the stairs or brisk walks), as the most enjoyable and easier to build into their daily routine. Strength based snacks and those which were more novel, such as lunges whilst vacuuming, were less popular, (Figs. 4, 5 and 6). The most frequently selected activity snacks that participants perceived that they would complete at home or during leisure time were aerobic activities including: a brisk walk in the park (41%), housework (37%), gardening (37%), climbing the stairs multiple times (27%) and run/walk/cycle to shops (26%). The most frequently reported activity snacks participants perceived they would complete at work included: taking the stairs (41%), brisk lunch time walk (31%) and walking to colleagues rather than calling/emailing (29%). Strength based activity snacks such as lunges at work (11%) and seated arm raises (8%) were seen as less preferable.

### Physical activity tracker and mobile app use

Most participants (90%) owned a smartphone and of those 45% used their phone to track their physical activity; of which 52% downloaded an app to their phone to allow tracking. Of those who used an app to track their physical activity, the most appealing features were the option to track steps (27%) and the ability to view a summary of their data (15%). A physical activity tracker or watch was owned by 35% of participants, and the Fitbit was the most commonly owned device (45%) of the 236 participants who completed the open text response. The three most appealing features of a physical activity tracker were notifications to move when sedentary for too long (16%), ability to track physical activity (15%), and the ease of use (15%).

## Discussion

This study investigated the acceptability of the Snacktivity™ concept with the public, as a novel approach to promoting small bouts of physical activity and reducing sedentary behaviour. Overall, Snacktivity™ was viewed positively by participants and findings highlighted that the public are open to different ways of achieving the recommended amount of physical activity each day. The small change approach has been shown to be effective in improving other health behaviours such as weight [12] and this study has provided further evidence to support such an approach for promoting physical activity.

### Interpretation of findings

Our findings showed that the majority liked the concept of Snacktivity™ and likeability was rated higher by females and those over 61 years of age. The flexibility and minimal time commitment associated with achieving the recommended doses of physical activity through Snacktivity™ were rated highly by responders. Findings provide evidence that a small changes approach such as Snacktivity™, is perceived as addressing the key barriers to physical inactivity in the population (e.g. perceived lack of resources and time) [13].

Our findings highlighted that Snacktivity™ may be more motivating to those who are physically inactive, those who are also most likely to benefit from increasing their physical activity and research shows small increases in physical activity are clinically important for the most inactive [20]. Furthermore, previous evidence suggests that the greatest gains to health are seen among the most physically inactive doing a little more activity per week, in comparison to the physically active doing even more [21].

Nearly 90% of participants perceived Snacktivity™ as easy to do on a typical non-working day and 59% on a typical working day. This variation between working and non-working days is noteworthy as an increase in working hours is associated with decreased physical activity [22] highlighting that work is often prioritised as more important to individuals than health behaviour change. This is particularly concerning given that those who were inactive and spent more hours of the day sitting had lower perceived ease of uptake of Snacktivity™ relative to active responders. Our findings further highlight the difficulties of increasing physical activity and reducing sedentary time in the population, even when the behavioural goal is broken into small bouts, such as in the case of Snacktivity™. Evidence from this study further emphasises the importance and necessity for the implementation of innovative approaches to physical activity such as Snacktivity™, to encourage those who are inactive to move more and be less sedentary during their working day.

Overall, more participants rated aerobic based activity snacks (i.e. brisk walks and taking the stairs) as the types of physical activity they would enjoy and could fit into their daily routine. In contrast, strength-based activity snacks and those that do not form part of usual routine (i.e. lunges and bicep curls) were less popular. These results highlight the need for physical activity campaigns and other behaviour change approaches to focus on the importance of muscle strengthening activities for health [23, 24], as well as aerobic based activities. Current guidance places greater emphasis upon the public achieving 150–300 min of moderate physical activity per week, and there is reduced focus on the importance of strength-based activities. Snacktivity™, which promotes both aerobic and strength-based activities, provides an opportunity to encourage the public to engage in both types of physical activity.

Snacktivity™ was disliked by 3% of our sample and a small proportion of responders noted that they did not like the term ‘Snacktivity™’. The most common reason reported by participants for disliking the concept was the concern that they may forget to regularly incorporate activity snacks into their routines. These concerns regarding ability to self-monitor and self-regulate health behaviour change were also echoed in reasons related to perceived uptake. Most participants reported that the support to self-monitor their behaviour would help them to do Snacktivity™ throughout their day. Consistent with other studies [25, 26], behaviour change techniques such as the ability to be challenged and motivated as well as techniques to encourage habit formation (i.e. alerts to mobile phone when sedentary and self-monitoring using a physical activity tracker) were also rated highly. Preferences for receiving alerts using technology, along with the fact that most of our sample own a smartphone, are noteworthy for the further development and practical application of Snacktivity™. Furthermore, just under half of study participants reported using their mobile phone to track their physical activity; a large proportion of which were accessing apps which they had downloaded onto their device. Findings suggest that providing methods to effectively self-monitor Snacktivity™ using a mobile phone app and technology, which are known to be effective for facilitating health behaviour change [27], could lead to sustained increase in physical activity among the general population.

### Implications

The promotion of physical activity has historically been encouraged via completion of long(er) bouts of activity, which has not been successful in getting the population more physically active. Our findings provide evidence for the acceptability of a novel and practical method to encourage the population to be less sedentary and move more on both working and non-working days through a small changes approach to promoting physical activity behaviour. Furthermore, preferences for the ability to receive notifications encouraging the completion of activity snacks and the option to self-monitor behaviour through technology should be considered for the further development and delivery of Snacktivity™.

### Strengths

To our knowledge, this is the first study to take a behavioural science perspective to exploring the acceptability of the Snacktivity™ approach. The study presents evidence for the acceptability of Snacktivity™ and provides useful insights and methods for translating guidance for physical activity into practice. We recruited participants across six general practices that varied in socio-economic deprivation, thus increasing the generalisability of the findings.

### Limitations

Physical activity status and sitting time were based upon self-reported recall and therefore should be interpreted with some caution. The response rate and the number of inactive participants was lower than expected. The number of active participants and those actively tracking their physical activity was high and therefore we cannot rule out the possibility that the study findings reflect the views of participants with more open and innovative views about health care and physical activity. However, it should also be noted that the lower than expected response rate may be a result of the survey being conducted during the COVID-19 pandemic. Furthermore, as the survey was distributed during the COVID-19 pandemic, the findings should be interpreted within this context as participants may have had a change in priorities, working practices or daily activities which may have impacted their views regarding physical activity.

## Conclusion

This study provides evidence that a small changes approach to increasing physical activity and reducing sedentary behaviour was viewed favourably and the Snacktivity™ approach to promoting physical activity appears acceptable to the public. Findings also provide indication of the preferred methods of delivery and implementation of such an approach using technology. Our findings highlight the need for interventions to be developed and tested in the population to assess whether Snacktivity™ can lead to sustained participation in physical activity. The World Health Assembly has set a target to reduce physical inactivity by 15% by 2030, and Snacktivity™ is one way in which this target may be achieved [28].

## Supplementary Information

**Additional file 1: Supplementary file 1**. Picture booklet illustrating activity snacks.

**Additional file 2: Supplementary file 2.** Snacktivity likeability data.

## Data Availability

The datasets used and analysed during this study are available from corresponding author on reasonable request. Access to anonymised data may be granted following review of the request. Exclusive use will be retained until the publication of major outputs from this research programme.

## Abbreviations

WHO: The World Health Organisation
GPPAQ: General Practice Physical Activity Questionnaire
IPAQ: International Physical Activity Questionnaire
MVPA: Moderate to vigorous physical activity
CRN: The Clinical Research Network
NHS: National Health Service
