# Supplementary figures and images for: Views of the public about Snacktivity™: a small changes approach to promoting physical activity and reducing sedentary behaviour
Source: BMC Public Health. 2022 Mar 29;22:618. doi: 10.1186/s12889-022-13050-x (PMC8964250; doi:10.1186/s12889-022-13050-x)

**
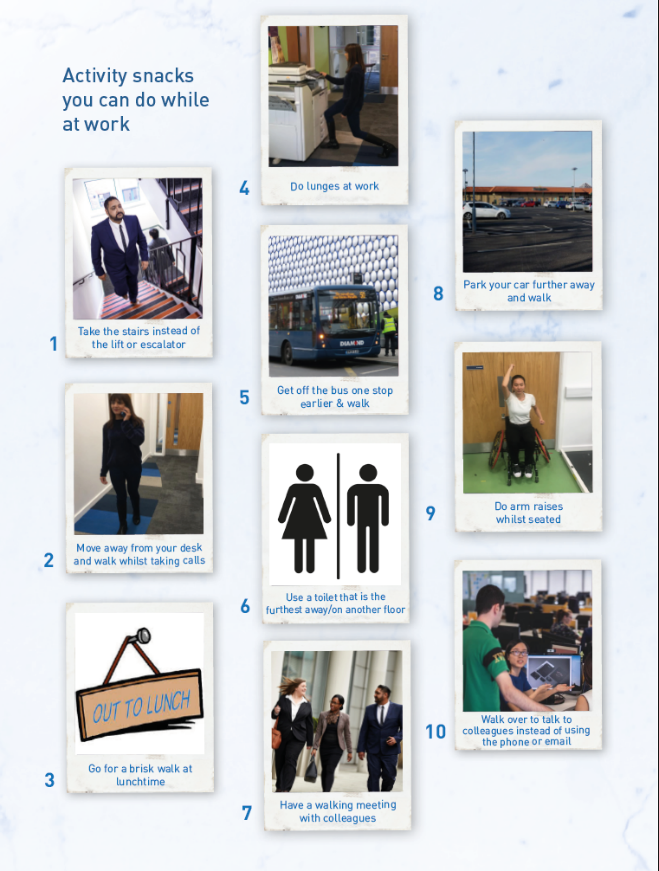
Supplementary file 1**: picture booklet illustrating activity snacks


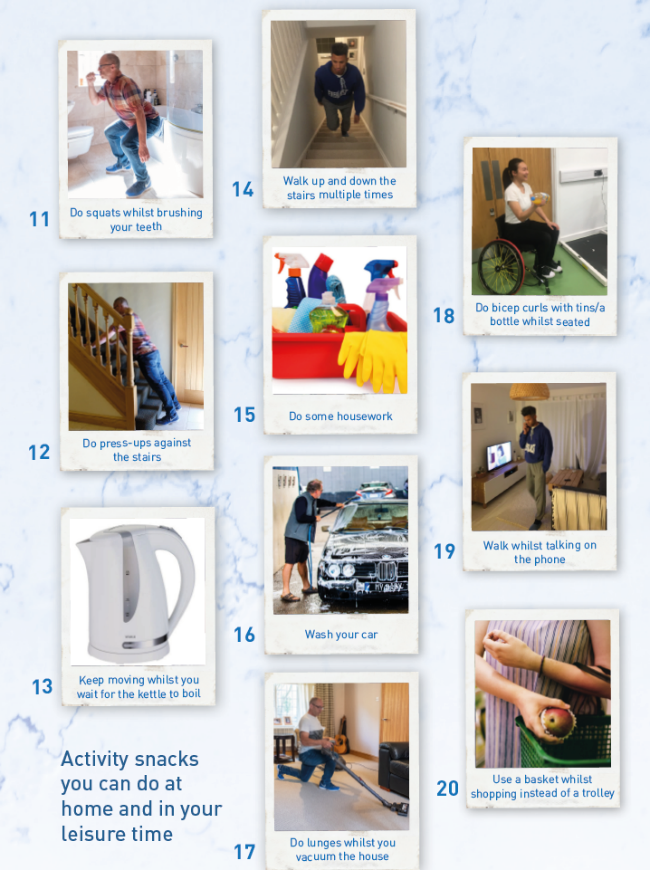


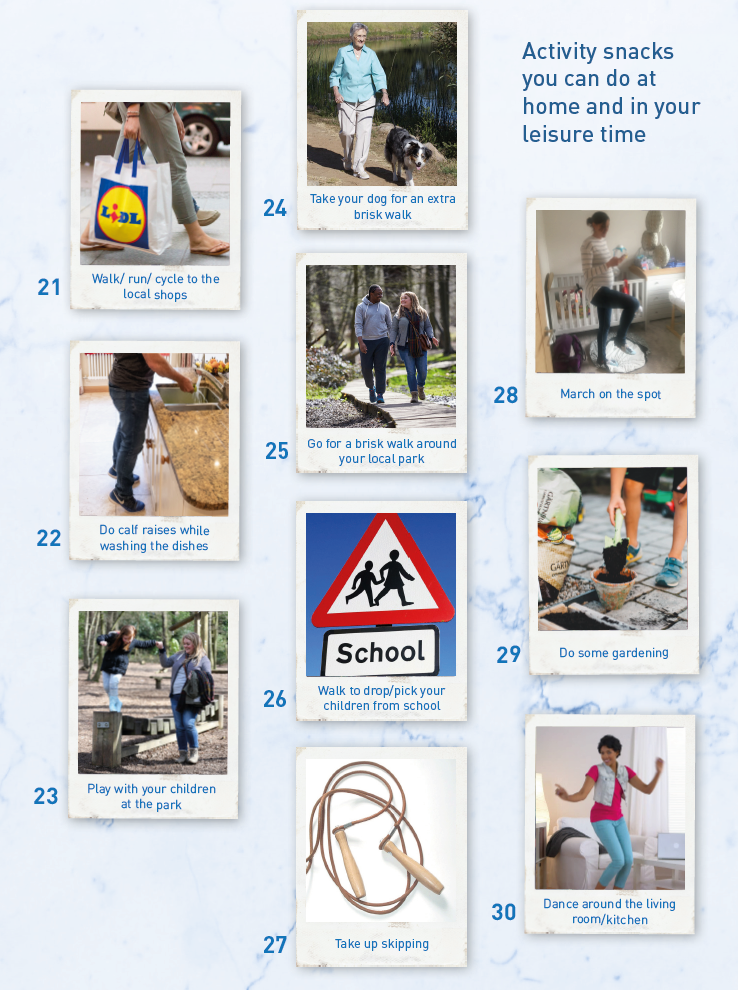

Supplement: Supplementary file 1 — Additional file 1: Supplementary file 1. Picture booklet illustrating activity snacks. [file 12889_2022_13050_MOESM1_ESM.docx]
