# Supplementary material for: Views of the public about Snacktivity™: a small changes approach to promoting physical activity and reducing sedentary behaviour
Source: BMC Public Health. 2022 Mar 29;22:618. doi: 10.1186/s12889-022-13050-x (PMC8964250; doi:10.1186/s12889-022-13050-x)
Supplement: Supplementary file 2 — Additional file 2: Supplementary file 2. Snacktivity likeability data. [file 12889_2022_13050_MOESM2_ESM.docx]

**Supplementary file 2:** Snacktivity likeability data

|  | | **All participants**  N=724 |
| --- | --- | --- |
| What are your thoughts about the idea of Snacktivity? | | |
| Like it a lot | | 366 (51%) |
| Like it a bit | | 240 (34%) |
| Neither like nor dislike | | 85 (12%) |
| Dislike it a bit | | 16 (2%) |
| Dislike it a lot | | 6 (<1%) |
| Missing | | 11 |
|  | | |
| **Model estimates^1^** | | **Odds ratio^2^ (95% CI), p-value** |
| Age (years) | ≤40 | 0.66 (0.46, 0.96), 0.03 |
|  | 41-60 | 1.11 (0.80, 1.53), 0.5 |
|  | ≥61 | REF |
| Gender | Male | REF |
|  | Female | 1.68 (1.25, 2.25), 0.001 |
|  | Prefer not to say | 0.82 (0.19, 3.48), 0.8 |
| Employment status | Employed | 1.11 (0.81, 1.52), 0.5 |
|  | Unemployed | REF |
|  | Other | 1.02 (0.46, 2.28), 1.0 |
| Socioeconomic Status | Low | REF |
|  | Medium | 1.07 (0.63, 1.81), 0.8 |
|  | High | 0.71 (0.47, 1.08), 0.1 |
| Physical activity status | Active | 0.77 (0.55, 1.08), 0.1 |
|  | Inactive | REF |
| Sitting time | <6 hours/day | 1.52 (0.93, 2.48), 0.1 |
|  | 6-10 hours/day | 1.15 (0.80, 1.65), 0.5 |
|  | >10 hours/day | REF |

^1^Estimates obtained from univariate models.

^2^Odds ratio values >1 indicate higher odds for liking Snacktivity than the reference group.
